# A Long-Term Study of Alignment Correction Following Proximal Femoral Varus Osteotomy and Pemberton Osteotomy in Children With Legg–Calvé–Perthes Disease and Developmental Dysplasia of the Hip

**DOI:** 10.3389/fped.2022.835447

**Published:** 2022-04-06

**Authors:** Kuei-Yu Liu, Kuan-Wen Wu, Chia-Che Lee, Sheng-Chieh Lin, Ken N. Kuo, Jia-Feng Chang, Ting-Ming Wang

**Affiliations:** ^1^Department of Medical Education, National Taiwan University Hospital Hsin-Chu Branch, Hsin-Chu, Taiwan; ^2^Department of Orthopaedic Surgery, National Taiwan University Hospital, Taipei, Taiwan; ^3^Department of Orthopaedic Surgery, Chung Shan Medical University Hospital, Taichung, Taiwan; ^4^Institute of Medicine, Chung Shan Medical University, Taichung, Taiwan; ^5^Cochrane Taiwan, Taipei Medical University, Taipei, Taiwan; ^6^Department of Internal Medicine, En Chu Kong Hospital, New Taipei City, Taiwan; ^7^Department of Orthopaedic Surgery, College of Medicine, National Taiwan University, Taipei, Taiwan

**Keywords:** femoral varus osteotomy, legg calve perthes disease, developmental dysplasia of hip (DDH), mechanical axis deviation, Pemberton osteotomy

## Abstract

Proximal femoral varus osteotomy (PFVO) is a common procedure performed in children with developmental dysplasia of the hip (DDH) and Legg–Calvé–Perthes disease (LCPD). However, the long-term effect on angular deformities of the knees and ankles following PFVO remains controversial. This study investigated the relationship between PFVO and alignment changes in the knee and ankle after the procedure. Twenty-five patients undergoing PFVO procedure with a minimum 4-year evaluation period were enrolled in the study, including 14 unilateral LCPD and 11 unilateral DDH. The standing scanogram examinations were collected before the operation, immediately following surgery, after a 1-year follow-up, after a 3-year follow-up, and at the final visit to the clinic. The radiographic parameters included leg length, femoral neck-shaft angle (FNSA), femorotibial angle (FTA), mechanical axis deviation (MAD), tibiotalar angle (TTA), and mechanical lateral distal femoral angle (mLDFA). At the final examination, FNSA demonstrated insignificant change between the operative and non-operative limbs in the DDH group. Compared with the postoperative result, FNSA significantly improved in the LCPD group (*p* = 0.039). Both groups did not develop statistical significance in TTA, mLDFA, MAD, and leg length discrepancy after more than a 5-year follow-up. From a biomechanical perspective that the foot passes more medial to the knee under the center of leg mass, varus knee was prone to develop. In order to correct the mechanical axis, the knee reverted to a valgus position gradually. Our study indicates that patients with LCPD or DDH receiving PFVO and Pemberton osteotomy narrow the gap of angular growth in knees and ankles between the operative and non-operative limbs after a long-term follow-up.

## Introduction

Proximal femoral varus osteotomy (PFVO) reduces articular pressure of the hip by enlarging the weight-bearing surfaces of the joint after turning the femoral head inward into the acetabulum (1). With a combination of PFVO and Pemberton or Dega osteotomy, improved hip joint congruency and remodeling can lead to a better outcome clinically and radiographically (2).

Unlike the hips, effects on the knee after PFVO or combined procedures have not reached a consensus. So far, there have only been a few reports making their speculations (3, 4). The knee was predisposed first into varus after PFVO and then reverted to a valgus position as suggested by one study (4). The valgus tendency of the knee to adjust the alternation of the mechanical axis was thought to be a compensation for the medial displacement of the femoral head following PFVO in another study (3).

Proximal femoral varus osteotomy attempts to correct excessive femoral anteversion in children with irreducible dislocations or subluxations when non-operative treatments fail in developmental dysplasia of the hip (DDH) and to better contain the femoral head in Legg–Calvé–Perthes disease (LCPD) (5–9). The speculation that valgus deformity of the knee may result from PFVO in LCPD has been controversial (3, 10, 11). A study reported that the mechanical axis deviation (MAD) had no significant difference after PFVO in LCPD (3). Another study suggested that LCPD treated with PFVO was predisposed to the development of genu valgum, but the angular deformity of the knee was still within the normal range (11). Similar to the relevance of PFVO in LCPD on the compensatory genu valgum, the association between PFVO in DDH and the angular deformity of the knee remained disputable. There was no study suggesting that the DDH itself causes genu valgum regardless of PFVO. To the best of our knowledge, there has been only one report with a number of patients showing significant discrepancies in the femorotibial angle (FTA) and at the intersection between the mechanical axis and the knee (4).

In this study, we hypothesized that PFVO combined with Pemberton osteotomy has no predisposition to knee or ankle malalignment on the operated side of patients with LCPD and DDH. Therefore, the objective of our study was to investigate the tendencies of remodeling showed in the femoral neck-shaft angle (FNSA) and the alignment change of the knee and ankle in both groups after the procedure.

## Materials and Methods

### Patients

At our institution, PFVO combined with Pemberton osteotomy was performed in 31 patients with unilateral LCPD between 2010 and 2020 and 17 patients with unilateral DDH between 2008 and 2018. Patients with LCPD under the age of 7 with extrusion of the femoral head or patients diagnosed with LCPD from ages 7 to 12 were recommended to undergo PFVO, and PFVO was recommended in patients with DDH diagnosed before 3 years old with failed non-operative treatment.

We set inclusion criteria of minimal follow-up of 48 months; therefore, 14 LCPD (13 boys and 1 girl) and 11 DDH (2 boys and 9 girls) were included in this study. All had the complete radiographic data preoperatively and postoperatively available for analysis. The patients who had additional surgeries within 4 years of follow-up were also excluded.

Among 14 LCPD, there were 7 patients with the right hip affected and 7 with the left hip affected. The mean age was 7.2 years (range, 4.0–10.7 years) at the operation and 12.7 years (range, 8.2–18.2 years) at the final examination. The mean postoperative follow-up period was 66.4 months (range, 48–105 months). Of 11 patients with DDH, the right hip was affected in 3 patients and the left in 8. The mean age was 6.9 years (range, 3.1–10.3 years) at the operation and 13.1 years (range, 8.8–21.0 years) at the final examination. The mean postoperative follow-up period was 74.9 months (range, 48–129 months) (Table 1).

**TABLE 1:** Baseline demographic features and operative characteristics of the patients in the study.

	Legg–Calvé–Perthes disease (*n* = 14)	Developmental dysplasia of the hip (*n* = 11)
Age at operation (years)	7.2 ± 2.0	6.9 ± 2.4
Age at final follow-up (years)	12.7 ± 2.8	13.1 ± 3.5
Follow-up (months)	66.4 ± 18.3	74.9 ± 23.6
Gender	Male 13; female 1	Male 2; female 9
Side involved	Left 7; right 7	Left 8; right 3
Weight (kg) at operation	30.0 ± 11.0	23.3 ± 6.0
Height (m) at operation	1.20 ± 0.12	1.19 ± 0.16
BMI (kg/m^2^) at operation	20.6 ± 5.8	16.2 ± 1.4

*BMI, body mass index.*

The radiographic examinations were taken before the operation, after the operation, after a 1-year follow-up, after a 3-year follow-up, and at the final visit to the clinic.

### Radiographic Examination

**FIGURE 1:**
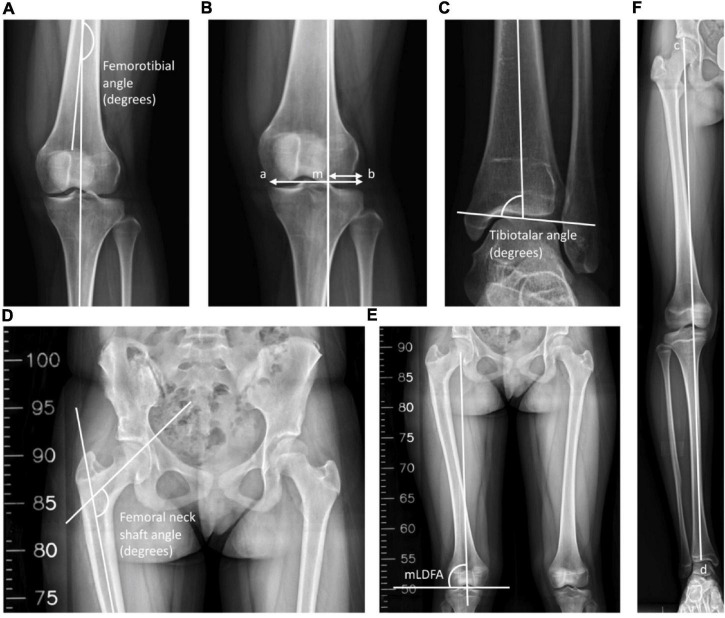
Radiological measurements. **(A)** The inferolateral angle between the anatomical axis of the femur and the tibia was defined as the femorotibial angle (FTA). **(B)** The mechanical axis of the leg was a line drawn through the center of the femoral head and the center of the tibial plafond, and M was the point of intersection between the knee and the mechanical axis. The mechanical axis deviation (MAD) was defined as the ratio of the distance between the mechanical axis and the lateral-most part of the tibia plateau to the width of the tibial plateau. MAD = (mb/ab) × 100%. **(C)** The superomedial angle between the anatomical axis of the tibia and the upper surface of the talus was defined as the tibiotalar angle (TTA). **(D)** The inferomedial angle between the longitudinal axis of the neck and shaft of the femur was defined as the femoral neck-shaft angle (FNSA). **(E)** The lateral angle between the mechanical axis of the femur and the lower surface of the femoral condyle was defined as the mechanical lateral distal femoral angle (mLDFA). **(F)** The leg length was defined as the distance between the top of the femoral head and the midpoint of the tibial plafond. Leg length = cd.

The radiographic examinations were taken before the operation, after the operation, after a 1-year follow-up, after a 3-year follow-up, and at the final visit to the clinic. All long-leg anteroposterior (AP) radiographs were taken at intervals in a standing position with the patella facing forward and both knees in full extension. The parameters of radiographic evaluation included the leg length, FNSA, FTA, MAD, tibiotalar angle (TTA), and mechanical lateral distal femoral angle (mLDFA) (Figure 1).

The leg length was labeled as the distance between the top of the femoral head and the midpoint of the tibial plafond. The angle between the femoral neck axis and the femoral shaft axis in the AP position was designated as the FNSA (12). The mLDFA was defined as the lateral angle between the mechanical axis of the femur and the lower surface of the femur, which connects the lowest points of the medial and lateral femoral condyle (13). We determined the MAD as the ratio of the distance between the mechanical axis and the lateral-most part of the tibia plateau to the width of the tibial plateau (14). A value < 50% was defined as valgus, and > 50% was defined as varus (14). The FTA was labeled as the angle between the anatomical axis of the femur and the tibia (15). Finally, the TTA was defined as the superomedial angle between the anatomical axis of the tibia and the superior articular surface of the talus (16). Angles of > 90° and < 90° are classified as valgus ankle and varus ankle, respectively (16, 17).

### Statistical Analysis

For the reliability test, the intraclass correlation coefficient (ICC) was used to analyze the intra-rater and inter-rater reliabilities of the FNSA, FTA, MAD, TTA, and mLDFA. For intra-rater reliability, the above measurements were repeated at a 1-week interval by the primary author (K-YL). Regarding inter-rater reliability, data were measured on separate occasions by two authors using the same method in Figure 1. The intra-rater ICCs for final FNSA, FTA, MAD, TTA, mLDFA, and leg length were 0.970, 0.901, 0.998, 0.979, 0.974, and 1.000, respectively; the inter-rater ICCs were 0.938, 0.849, 0.990, 0.944, 0.915, and 0.999, respectively.

The statistical analysis was performed using Student’s t-test when comparing variables showing normal distribution, and the chi-squared test was used for the categorical data. For all analyses, the statistical significance was defined as *p* < 0.05.

## Results

### Legg–Calvé–Perthes Disease With Proximal Femoral Varus Osteotomy

**FIGURE 2:**
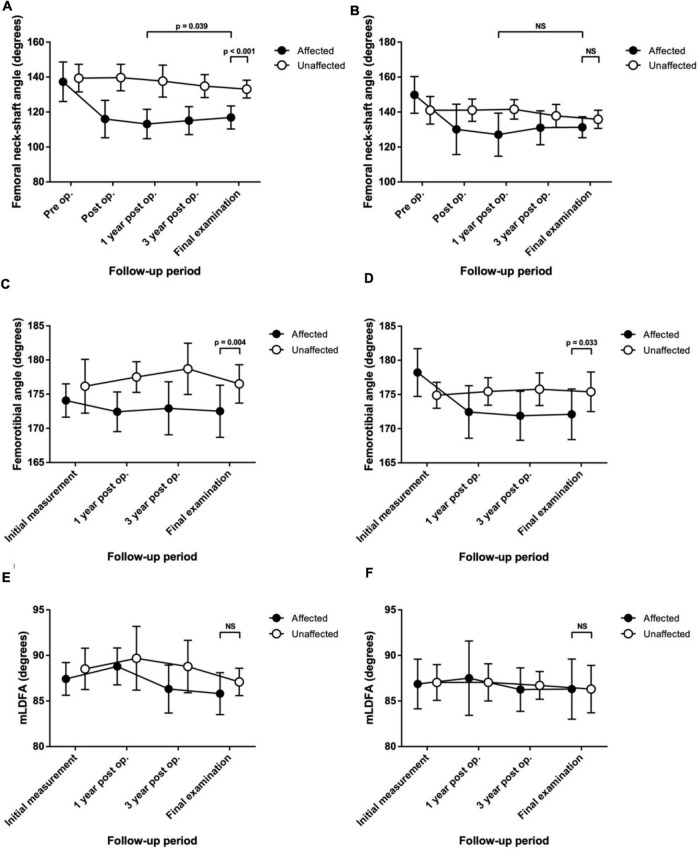
Serial change in the radiological measurements up to the final assessment in patients with Legg–Calvé–Perthes disease (LCPD) or developmental dysplasia of the hip (DDH) who were treated with proximal femoral varus osteotomy (PFVO) and Pemberton osteotomy. **(A)** Femoral neck-shaft angle of patients with LCPD. **(B)** Femoral neck-shaft angle of patients with DDH. **(C)** Femorotibial angle of patients with LCPD. **(D)** Femorotibial angle of patients with DDH. **(E)** Mechanical lateral distal femoral angle (mLDFA) of patients with LCPD. **(F)** mLDFA of patients with DDH. NS, not significant.

The differences in the FNSA between both sides were 2.1° ± 7.6° preoperatively, 23.8° ± 10.2° postoperatively, 24.6° ± 10.5° at a 1-year follow-up, 19.8° ± 9.9° at a 3-year follow-up, and 16.3° ± 9.4° at the final examination with statistical significance (*p* < 0.001) (Figure 2A). The mean FNSA on the affected side was 113.2° ± 8.4° after 1 year and 116.9° ± 6.6° at the final measurement with statistical significance (*p* = 0.039). The final mean FTA was 172.5° ± 3.8° on the affected side and 176.5° ± 2.8° on the normal side, with a statistically significant difference (*p* = 0.004) (Figure 2C).

**FIGURE 3:**
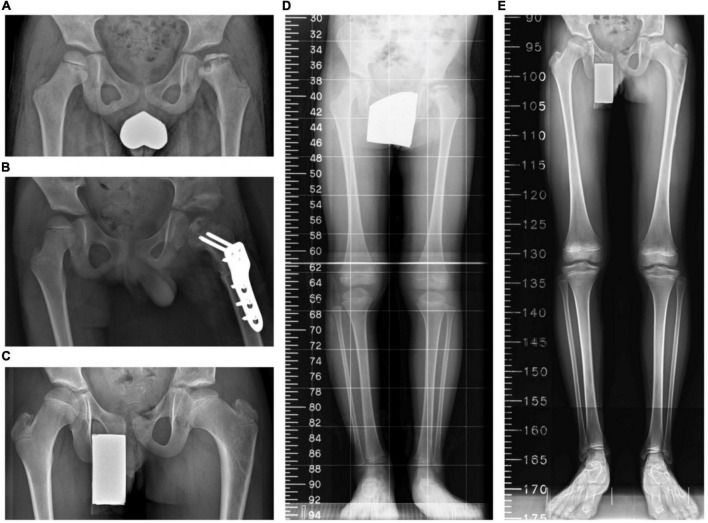
A boy aged 6 years 4 months with Legg–Calvé–Perthes disease (LCPD) who underwent the index procedures. **(A)** A preoperative standing anteroposterior pelvis view. **(B)** A postoperative anteroposterior pelvis view. **(C)** The latest standing anteroposterior pelvis view 76 months after the operation. **(D)** A preoperative scanogram. **(E)** The latest scanogram 76 months after the operation.

By contrast, the distribution of patients with knee malalignment on the affected (operative) and unaffected (non-operative) sides showed no significant difference eventually. On the affected limb, six patients developed genu varum (MAD > 50%), and 8 patients showed genu valgum (MAD < 50%). On the unaffected limb, 6 patients had MAD > 50%, and 8 patients progressed to MAD < 50%. Similarly, there was no significant difference in the tendency to develop valgus or varus ankle. There were more patients with TTA > 90° than TTA < 90° (11 versus 3, respectively) on the operative side. As to the normal side, there were 11 patients with TTA > 90° and 3 patients with TTA < 90°. Likewise, the mLDFA values of the affected limbs were not distinguishable from those of the unaffected limbs (85.8° ± 2.3° versus 87.1° ± 1.5°, *p* = 0.081) (Figure 2E). The performed varus angulation did not correlate well with the FTA, TTA, mLDFA, and MAD difference (final - preop). The final difference in leg length was 0.7 ± 0.9 cm with no significant difference (*p* = 0.798). The final radiographic measurements of the patients with LCPD are shown in Table 2. Serial radiographs of a patient with LCPD of the left hip underwent varus osteotomy and Pemberton procedure with 76-month follow-up are demonstrated in Figure 3.

**TABLE 2:** Differences in the radiographic examination in the affected and unaffected limbs of patients with LCPD at the final assessment (mean ± SD).

	Affected side	Unaffected side	*p*-Value
Femoral neck-shaft angle (degrees)	116.9 ± 6.6	133.1 ± 5.1	<0.001
Femorotibial angle (degrees)	172.5 ± 3.8	176.5 ± 2.8	<0.05
Tibiotalar angle (degrees)	93.4 ± 4.7	94.0 ± 4.8	0.743
mLDFA (degrees)	85.8 ± 2.3	87.1 ± 1.5	0.081
Mechanical axis deviation (%)	48.0 ± 14.9	54.4 ± 12.1	0.221
Leg length (cm)	75.0 ± 6.9	75.7 ± 7.6	0.798

*mLDFA, mechanical lateral distal femoral angle; LCPD, Legg–Calvé–Perthes disease.*

### Developmental Dysplasia of the Hip With Proximal Femoral Varus Osteotomy

**FIGURE 4:**
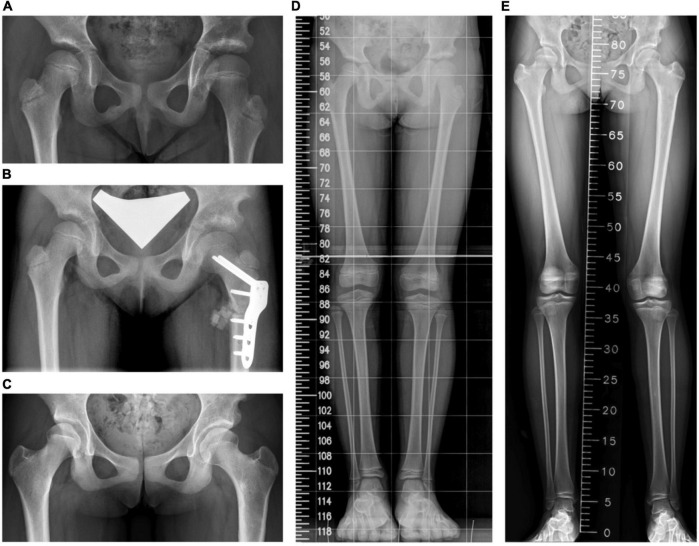
A girl who had open reduction and Pemberton procedure for left hip developmental dysplasia of the hip (DDH) earlier underwent the proximal femoral osteotomy for coxa valga. **(A)** A preoperative standing pelvis anteroposterior view. **(B)** A postoperative standing pelvis anteroposterior view. **(C)** The latest standing pelvis anteroposterior view 61 months after the operation. **(D)** A preoperative scanogram. **(E)** The latest scanogram 61 months after the operation.

The final mean FNSA was 131.3° ± 6.0° on the affected side and 135.8° ± 5.2° on the normal side, and the difference was not statistically significant (*p* = 0.074) (Figure 2B). The mean FNSA on the affected side was 127.1° ± 12.3° after 1 year and 131.3° ± 6.0° at the final measurement without statistical significance (*p* = 0.168). Regarding the FTA, the distinction between both sides at the final examination was statistically significant (172.1° ± 3.7° on the affected side and 175.8° ± 2.4° on the normal side, *p* = 0.033) (Figure 2D). Similar to the results of LCPD, the mLDFA of the operative limbs was not distinct from that of the normal limbs (86.3° ± 3.3° versus 86.3° ± 2.6°, *p* = 0.961) (Figure 2F). Furthermore, the results of MAD and TTA did not show knee or ankle malalignment. There were fewer patients with MAD > 50% than MAD < 50% (5 versus 6, respectively) on the operative side. As to the normal side, there were 7 patients with MAD > 50% and 4 patients with MAD < 50%. On the affected limb, 8 patients developed valgus ankle (TTA > 90°), and 3 patients showed varus ankle (TTA < 90°). On the unaffected limb, 6 patients had the TTA > 90°, and 5 patients progressed to TTA < 90°. Resembling the results of LCPD, the performed varus angulation did not correlate well with the FTA, TTA, mLDFA, and MAD difference (final - preop). The final difference in leg length was 0.1 ± 0.8 cm with no significant difference (*p* = 0.983). The final radiographic measurements of the patients with DDH are shown in Table 3. Figure 4 demonstrates a 5-year follow-up after surgery in a child with left hip DDH who underwent PFVO and Pemberton procedure.

**TABLE 3:** Differences in the mean radiographic examination in the affected and unaffected limbs of patients with DDH at the final assessment (mean ± SD).

	Affected side	Unaffected side	*p*-Value
Femoral neck-shaft angle (degrees)	131.3 ± 6.0	135.8 ± 5.2	0.074
Femorotibial angle (degrees)	172.1 ± 3.7	175.4 ± 2.9	<0.05
Tibiotalar angle (degrees)	92.2 ± 3.9	89.5 ± 3.0	0.090
mLDFA (degrees)	86.3 ± 3.3	86.3 ± 2.6	0.961
Mechanical axis deviation (%)	48.4 ± 17.9	53.5 ± 11.0	0.432
Leg length (cm)	76.0 ± 6.2	76.1 ± 6.7	0.983

*mLDFA, mechanical lateral distal femoral angle; DDH, developmental dysplasia of the hip.*

### Excluded Patient Due to Short Follow-Up Period

The short follow-up period of less than 48 months was mostly due to the second surgery for leg length discrepancy (LLD), MAD of non-operated side, and femoral greater trochanteric overgrowth deformity. Of the 17 excluded patients with LCPD, three received growth tethering for LLD on the operated side of PFVO, nine underwent greater trochanteric epiphysiodesis for greater trochanteric overgrowth on the operated side of PFVO, and two had guided growth for MAD on the non-operated side of PFVO. Regarding the four excluded patients with DDH, growth tethering for LLD was performed on the operated side of PFVO in all of them before 4 years of follow-up. None of the patients who required additional surgery of the lower extremities had any correlation with knee valgus deformity.

## Discussion

Proximal femoral varus osteotomy creates a better containment of the femoral head in LCPD, and it corrects excessive femoral anteversion in children with DDH. A comprehensive understanding of the procedure may help prevent residual problems. Developing angular deformities of the knee in patients with LCPD or DDH following PFVO has long been controversial. This retrospective cohort study was initially aimed at questioning the ambiguity of limb alignment change after PFVO in patients with LCPD and DDH. We, then, further assessed the effects of PFVO combined with Pemberton osteotomy on FNSA, FTA, TTA, mLDFA, MAD, and leg length. For patients with a minimum 4-year follow-up, FNSA demonstrated statistical significance in LCPD but not DDH. Both groups of patients with LCPD or DDH did not develop statistical significance in TTA, mLDFA, MAD, and LLD after a minimum 4-year follow-up. As to those excluded patients in this study, the qualitative results can remind surgeons of the possibility that patients with LCPD or DDH may still require further surgical procedures after the initial operation.

The limitations of this study were a small number of patients and only medium time of follow-up. However, in this study, all procedures were performed by one senior author (T-MW), resulting in less inter-surgeon variability. The previously published data from other institutions included multiple orthopedic surgeons performing the surgeries (3, 4, 10, 11). There is possible variability of surgeons’ preferences and different stages of experiences and their learning curves. Besides, compared to other patients in studies with a single procedure, all patients in this study underwent PFVO and Pemberton osteotomy, which was supposed to lead to a better outcome of the hip joint congruency (2, 3, 10, 11). The combined procedure is likely to lessen the impact of non-uniform stress distribution on the incongruence of the articular surfaces.

Overall, this study demonstrated that patients with LCPD or DDH who received PFVO showed a similar distribution of knee valgus alignment in both affected and unaffected limbs. Our results of MAD in LCPD concur with those of Kitakoji et al. (3) on their study of 30 patients but are in conflict with those of Tercier et al. (11) on their study of 33 patients (3, 11). Glard et al. (10) showed that the distribution of knee valgus had no significant difference between the operative and non-operative groups in 28 patients, and they suggested that the angular deformity of the knee was related to LCPD itself (10). As to DDH, our data on MAD showed no statistical significance between operated and non-operated sides, whereas one Japanese study involving 19 patients with a mean follow-up of 15.4 years demonstrated significant differences in MAD between both limbs (4). Moreover, the Japanese study and our study both showed a smaller FTA and MAD on the operated side compared to the non-operated side (4). From a biomechanical perspective, it was first found that varus knee tended to develop since the leg center of mass to the foot passes more medially to the knee, and it reverted to a valgus position gradually in order to correct the mechanical axis (2–4).

In our search, there was no previous literature available on ankle angular position alteration following PFVO. Since the standing radiographs were available before and after surgery, we had measured the ankle joint varus and valgus angles. The results showed that there was no correlation of PFVO to subsequent ankle positions.

The results of FNSA on the operated side in patients with LCPD showed a remodeling process in our research; however, the same tendency was not observed in our patients with DDH. Previous reports demonstrated that remodeling of approximately two-thirds of the original FNSA angulation occurred after PFVO for treating LCPD, and it was independent of the patient’s age at presentation (18, 19). Besides, the remodeling continued up to skeletal maturity (20). We supposed that the remodeling, which corrects the angular deformity at the osteotomy side, can somewhat explain the circumstance happening in LCPD according to Wolff’s law, where new bone increases on the concave side of the osteotomy and is reabsorbed in the convex side of the fracture (18, 21–23). As to DDH, the remodeling angles had a strong negative correlation with the postoperative FNSA (4). In our cases, the initial varus angle was more with LCPD vs. DDH (113.2° vs. 127.1°), which might be the reason that LCPD cases had faster remodeling. Although it was suggested that almost every case of remodeling came about in the first 6 years, a longer follow-up period may be required to observe such a phenomenon (4, 22).

In conclusion, we demonstrated that patients with LCPD or DDH who received PFVO and Pemberton osteotomy had a narrow gap in angular growth in knees and ankles between the operative and non-operative limbs after a long-term follow-up. In light of the potential of the index procedure to bridge the gap, additional surgical intervention may not be mandatory for LCPD and DDH to avoid harmful effects on a child’s growth and development.

## Data Availability Statement

The raw data supporting the conclusions of this article will be made available by the authors, without undue reservation.

## Ethics Statement

The studies involving human participants were reviewed and approved by Research Committee C, National Taiwan University Hospital. Written informed consent to participate in this study was provided by the participants’ legal guardian/next of kin.

## Author Contributions

All authors listed have made a substantial, direct, and intellectual contribution to the work, and approved it for publication.

## Conflict of Interest

The authors declare that the research was conducted in the absence of any commercial or financial relationships that could be construed as a potential conflict of interest.

## Publisher’s Note

All claims expressed in this article are solely those of the authors and do not necessarily represent those of their affiliated organizations, or those of the publisher, the editors and the reviewers. Any product that may be evaluated in this article, or claim that may be made by its manufacturer, is not guaranteed or endorsed by the publisher.

## References

[1] Pauwels F. *Biomechanics of the Normal and Diseased Hip: Theoretical Foundation, Technique and Results of Treatment An Atlas.* Berlin: Springer-Verlag (1976). 148 p.

[2] Kamegaya M Morita M Saisu T Kakizaki J Oikawa Y Segawa Y. Single versus combined procedures for severely involved legg-calvé-perthes disease. *J Pediatr Orthop.* (2018) 38:312–9. 10.1097/BPO.0000000000000840 27442215

[3] Kitakoji T Hattori T Iwata H. Femoral varus osteotomy in legg-calvé-perthes disease: points at operation to prevent residual problems. *J Pediatr Orthop.* (1999) 19:76–81. 10.1097/01241398-199901000-00017 9890292

[4] Suda H Hattori T Iwata H. Varus derotation osteotomy for persistent dysplasia in congenital dislocation of the hip. proximal femoral growth and alignment changes in the leg. *J Bone Joint Surg Br.* (1995) 77:756–61. 10.1302/0301-620x.77b5.7559705 7559705

[5] Canario AT Williams L Wientroub S Catterall A Lloyd-Roberts GC. A controlled study of the results of femoral osteotomy in severe Perthes’ disease. *J Bone Joint Surg Br.* (1980) 62-B:438–40. 10.1302/0301-620X.62B4.7430219 7430219

[6] Dezateux C Rosendahl K. Developmental dysplasia of the hip. *Lancet.* (2007) 369:1541–52. 10.1016/S0140-6736(07)60710-7 17482986

[7] Herring JA. The treatment of legg-calvé-perthes disease. a critical review of the literature. *J Bone Joint Surg Am.* (1994) 76:448–58. 10.2106/00004623-199403000-00017 8126052

[8] Salter RB. The present status of surgical treatment for legg-perthes disease. *J Bone Joint Surg Am.* (1984) 66:961–6. 10.2106/00004623-198466060-00021 6736099

[9] Vitale MG Skaggs DL. Developmental dysplasia of the hip from six months to four years of age. *J Am Acad Orthop Surg.* (2001) 9:401–11. 10.5435/00124635-200111000-00005 11730331

[10] Glard Y Katchburian MV Jacquemier M Guillaume JM Bollini G. Genu valgum in legg-calvé-perthes disease treated with femoral varus osteotomy. *Clin Orthop Relat Res.* (2009) 467:1587–90. 10.1007/s11999-009-0727-8 19214643 PMC2674167

[11] Tercier S Shah H Siddesh ND Joseph B. Does proximal femoral varus osteotomy in legg-calvé-perthes disease predispose to angular mal-alignment of the knee? A clinical and radiographic study at skeletal maturity. *J Child Orthop.* (2013) 7:205–11. 10.1007/s11832-013-0487-6 24432079 PMC3672457

[12] Armbuster TG Guerra J Jr. Resnick D Goergen TG Feingold ML Niwayama G The adult hip: an anatomic study. Part I: the bony landmarks. *Radiology.* (1978) 128:1–10. 10.1148/128.1.1 663192

[13] Paley D. *Principles of Deformity Correction.* Heidelberg: Sringer-Verlag (2002). 8 p.

[14] Paley D Tetsworth K. Mechanical axis deviation of the lower limbs. preoperative planning of uniapical angular deformities of the tibia or femur. *Clin Orthop Relat Res.* (1992) 280:48–64. 1611764

[15] Brouwer GM van Tol AW Bergink AP Belo JN Bernsen RM Reijman M Association between valgus and varus alignment and the development and progression of radiographic osteoarthritis of the knee. *Arthritis Rheum.* (2007) 56:1204–11. 10.1002/art.22515 17393449

[16] Knupp M Ledermann H Magerkurth O Hinterman B. The surgical tibiotalar angle: a radiologic study. *Foot Ankle Int.* (2005) 26:713–6. 10.1177/107110070502600909 16174502

[17] Davids JR Gibson TW Pugh LI. Quantitative segmental analysis of weight-bearing radiographs of the foot and ankle for children: normal alignment. *J Pediatr Orthop.* (2005) 25:769–76. 10.1097/01.bpo.0000173244.74065.e4 16294134

[18] Herceg MB Cutright MT Weiner DS. Remodeling of the proximal femur after upper femoral varus osteotomy for the treatment of legg-calvé-perthes disease. *J Pediatr Orthop.* (2004) 24:654–7. 10.1097/00004694-200411000-00012 15502566

[19] Talkhani IS Moore DP Dowling FE Fogarty EE. Neck-shaft angle remodelling after derotation varus osteotomy for severe perthes disease. *Acta Orthop Belg.* (2001) 67:248–51. 11486687

[20] Shah H Siddesh ND Joseph B. To what extent does remodeling of the proximal femur and the acetabulum occur between disease healing and skeletal maturity in Perthes disease? A radiological study. *J Pediatr Orthop.* (2008) 28:711–6. 10.1097/BPO.0b013e31818456dc 18812895

[21] Stilli S Magnani M Lampasi M Antonioli D Bettuzzi C Donzelli O. Remodelling and overgrowth after conservative treatment for femoral and tibial shaft fractures in children. *Chir Organi Mov.* (2008) 91:13–9. 10.1007/s12306-007-0003-6 18320368

[22] Wallace ME Hoffman EB. Remodelling of angular deformity after femoral shaft fractures in children. *J Bone Joint Surg Br.* (1992) 74:765–9. 10.1302/0301-620X.74B5.1527131 1527131

[23] Wolff J. *The Law of Bone Remodelling.* Berlin: Springer-Verlag (1986). 1 p.

